# Hole-in-one: simple non-surgical technique for the management of anterior chamber migrated Ozurdex^®^ implant

**DOI:** 10.3205/oc000132

**Published:** 2020-02-27

**Authors:** Pablo Rivera-Pérez de Rada, Pedro Fernández-Avellaneda, Lucía Teresa Barturen Herraiz, Iker Henares Fernández, Estibaliz Ispizua Mendivil, Maria Ángeles Castellanos Relloso, Javier Hidalgo-Santamaría, Jesús Alfonso Grijalvo López

**Affiliations:** 1 Department of Ophthalmology, Basurto University Hospital, Bilbao, Spain; 2 Madrid, Spain; 3 Faculty of Medicine and Nursing, University of the Basque Country (UPV/EHU), Lejona, Vizcaya, Spain

**Keywords:** dexamethasone, macular edema, pupil, anterior chamber, piilocarpine, intravitreal injections, corneal endothelium, posterior eye segment, hospital emergency service

## Abstract

**Introduction:** The migration of a dexamethasone implant to the anterior chamber is a vision-threatening complication which can happen in non-compartmentalized eyes treated with this device. Previous literature suggests that the solution to this complication is almost always surgical and in most cases cannot be delayed.

**Case description:** We present the case of a 78-year-old woman with a scleral-fixated IOL and macular edema treated with Ozurdex^®^. She came to us complaining of blurred vision and was subsequently diagnosed with an anterior-chamber migration of her dexamethasone implant. Postural manoeuvres were performed until the dexamethasone implant returned to the vitreous cavity through the pupil. Pilocarpine drops were prescribed with a positive outcome and no further migrations were described.

**Discussion:** This case shows a practical and efficient way of managing a potentially vision-threatening complication without placing the patient onto an operating table. It is interesting to see how it is possible to relocate a dexamethasone implant despite the presence of a scleral-fixated IOL.

**Conclusion:** Postural manoeuvres are an interesting option in patients with a dexamethasone implant migrated to the anterior chamber. This approach can have very positive outcomes, in addition to avoiding surgery, with all the risks and complications involved.

## Introduction

Ozurdex^®^ (Allergan, USA) is a sustained-release dexamethasone implant used to treat various vitreoretinal diseases [1]. A challenging complication of the intravitreal injection of Ozurdex^®^ is the migration to the anterior chamber in decompartmentalized patients. Reported first time by Pardo-López et al. in 2012 [2], Ozurdex^®^ migration to the anterior chamber requires an immediate resolution, as it can cause permanent endothelial damage to the cornea [3], [4]. Speed in treating this complication is the key for a good prognosis, since taking a long time before removing the implant from the anterior chamber is associated with a worse endothelial account and lower visual acuity [4].

## Case description

We present the case of a 78-year-old woman with a pseudoexfoliative syndrome. She had been operated for cataracts in both eyes, without incident. Five years later, her left eye developed IOL-capsular bag complex subluxation, for which she was operated again, fixing the IOL-capsular bag complex to the sclera with nylon suture (SFIOL). Afterwards, she developed macular edema and received an intravitreal injection of Ozurdex^®^. 

Three weeks after the injection, she came to the emergency room complaining of “blurred vision”. Visual acuity was 10/200. The slit lamp examination confirmed the presence of the implant in the inferior angle of the anterior chamber, with corneal edema associated. 

**Figure 1:**
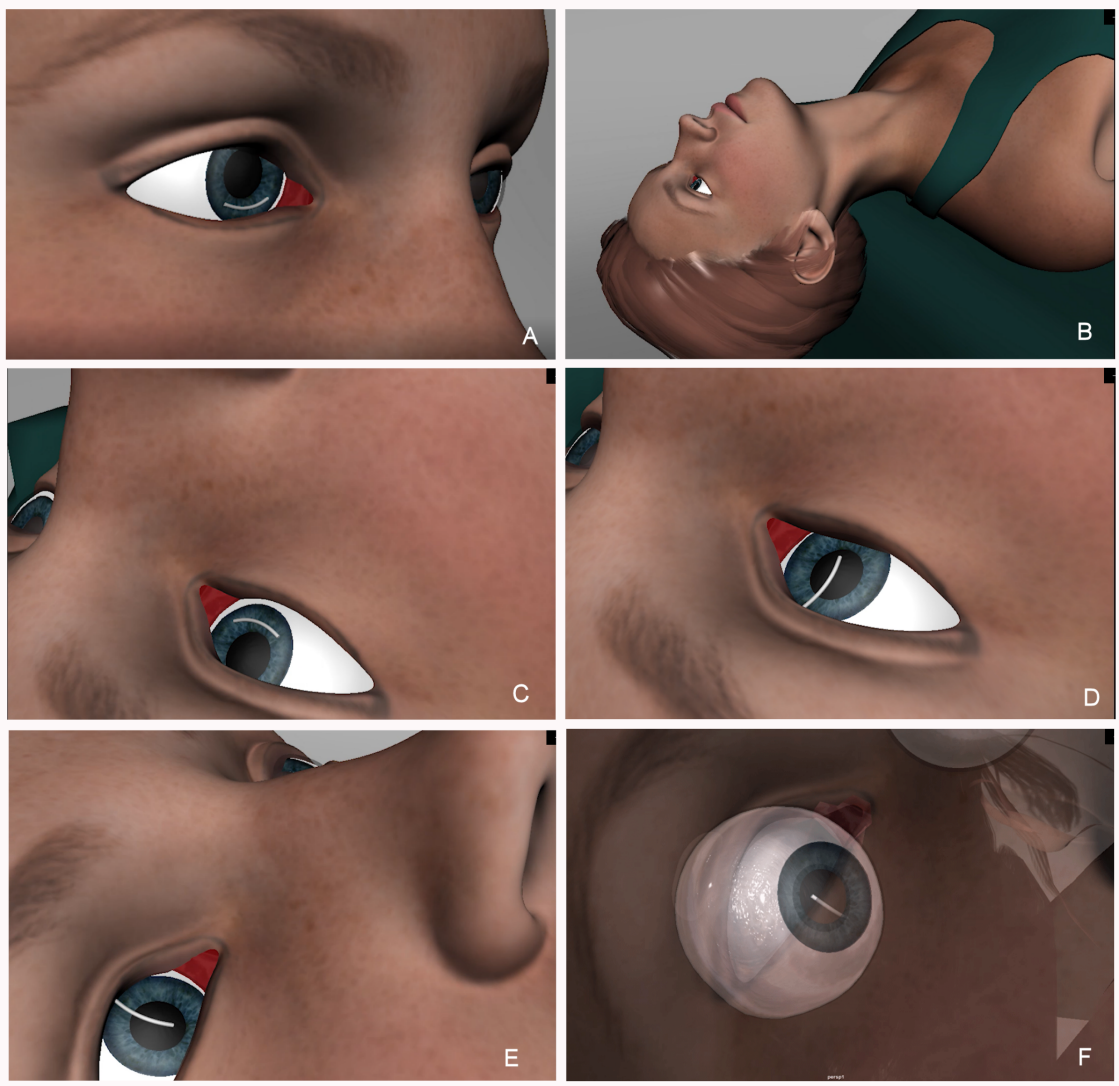
A) Ozurdex^®^ located in the inferior angle. B) The patient lies in supine, with the head leaning out. C) At first, the implant does not move. D) With gentle massage, the implant moves to the superior angle, in vertical position. E) The patient’s head is elevated so the inferior tip of the implant can pass under the pupil. F) When the patient looks down, the implant moves back to the vitreous chamber.

**Figure 2:**
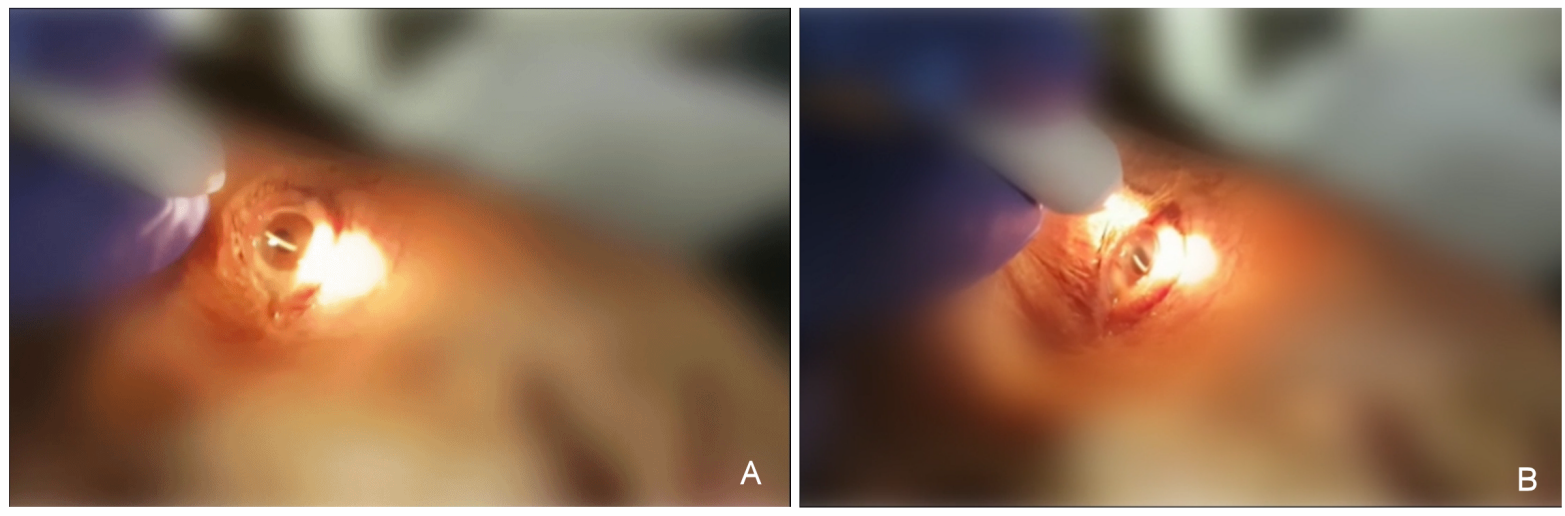
The images show the actual moment when the dexamethasone implant moves from the anterior to the vitrous chamber through the pupil. A) Looking up, the implant moves to the superior angle. B) Looking down, the implant migrates back.

At that moment, postural relocation was the course of action that was decided upon. The patient’s pupil was dilated with tropicamide and phenylephrine to the maximum dosage. Even so, dilation was medium-low. The patient was placed in a stretcher, lying in a supine position with her head leaning out, and she was asked to look up to her hair. That way, the dexamethasone implant could move from the inferior to the superior angle. At first, the implant did not move, as it was adhered to the structures of the inferior angle. Topical anaesthesia was instilled and a gentle massage of the globe was performed. After thirty seconds, the implant moved from the inferior to the superior angle, changing from horizontal to vertical position. After that, the patient’s head was elevated to facilitate the inferior tip of the implant to pass under the pupil and then the whole implant entered the vitreous chamber by asking the patient to look down (Figure 1 (Fig. 1), Figure 2 (Fig. 2)).

Pilocarpine drops were prescribed and the patient was instructed to avoid prone position. She was closely followed for one year, with good IOP control. Corneal endothelium cell count was of 2,167 cells in her right eye and 547 cells in her left eye, which was the one that underwent the aforementioned complication. The implant did not migrate again and visual acuity remained stable in 20/100. 

## Discussion

The treatment options for Ozurdex^®^ migration to the anterior chamber are various: surgical extraction with forceps, surgical extraction with aspirator, surgical extraction with viscoelastic, surgical relocation to the vitreous chamber with viscoelastic, and non-surgical postural relocation [4], [5], [6], [7], [8]. For this last option, very little has been published. 

In articles concerning treatment for anterior chamber migrated dexamethasone implants, postural management is rarely mentioned. The technique is usually described as “dilating the pupil and advising supine positioning” and it has been proven to work in certain cases. 

One of the most interesting points about this case is the fact that the implant correctly relocated to the vitreous chamber even though there was an SFIOL. To our knowledge, this is the second time [6] in the published literature that postural treatment is used to treat a patient with SFIOL and a dexamethasone implant migrated to the anterior chamber. 

Surgical extraction of an Ozurdex^®^ implant presents several problems: Firstly, the patient was treated with a dexamethasone implant because of vitreoretinal pathology. By extracting it, we would extract a therapeutic medication. Moreover, the extraction surgery, as with any other surgery, is injurious to the corneal endothelium and therefore can worsen endothelium cell count [3]. It is also worth noting that during the surgery, the dexamethasone implant can be disrupted due to the manipulation and subsequent fragmentation into many pieces [3]. Also, an operating theatre with a microscope, proper surgical tools, and a fully trained anterior segment surgeon is required. 

## Conclusion

Relocating the implant instead of extracting it can occasionally be a good approach. It presents some advantages: the difficulty of a complete extraction is avoided, as is another intravitreal injection which reduces treatment costs. If the relocation process is carried out with postural manoeuvres instead of surgery, it is possible to resolve the case in the emergency room and treatment is not delayed hours or days until an operating room is made available.

## Audiovisual material

A 3D reconstruction of the technique is included, as well as an actual video of the procedure (Attachment 1). 

## Notes

### Author contributions

- Rivera-Pérez de Rada, Pablo: treatment, writing: original draft, writing: review and editing
- Fernández-Avellaneda, Pedro: writing: review and editing
- Barturen Herraiz, Lucía Teresa: diagnosis, treatment, writing: review and editing
- Henares Fernández , Iker: treatment, writing: review and editing
- Ispizua Mendívil, Estíbaliz: writing: review and editing
- Castellanos Relloso, María Ángeles: diagnosis, writing: review and editing
- Hidalgo-Santamaría, Javier: 3D video artist
- Grijalvo López, Jesús Alfonso: writing: review and editing

### Informed consent 

The authors certify that they have obtained all written, appropriate patient consent forms. In the form, the patient has given her consent for her images and other clinical information to be reported in a medical journal. The patient understands that her name and initials will not be published, but although efforts will be made to conceal her identity, anonymity cannot be guaranteed. 

### Competing interests

The authors declare that they have no competing interests.

## Supplementary Material

Video: 3D reconstruction of the technique postoral recolocation with Ozurdex®
